# Coronavirus 2020: From lockdown to resumption at the National Library of Scotland

**DOI:** 10.1177/0955749020983478

**Published:** 2020-08

**Authors:** John Scally

**Affiliations:** 47758 National Library of Scotland, Edinburgh, UK

**Keywords:** coronavirus, crisis, digital library, government, homeworking, libraries, national libraries, pandemic, social media, well-being

## Abstract

Pandemics are nothing new to libraries. Our collections contain many works on epidemics and pestilence through the centuries. The Covid-19 pandemic forced the National Library of Scotland to deal with a public health crisis that went beyond the experience and policies of the institution. This account describes the measures taken to close the Library rapidly and to set up a staff of over 300 to homeworking. It is then explained how the Library continued working under lockdown and provided a digital service to the public. In the final section, the Library’s approach to reoccupying the buildings and resuming public services is discussed, emphasising the complex administrative and human issues involved. Finally, a short assessment of the ‘new normal’ for the National Library is posited.

## Introduction

Until the start of 2020, pandemics were events that librarians assisted readers with finding resources about in the collections. The plague of Justinian (541–542), the Black Death (1346–1353) and, in the last century, Spanish Flu (1918–1920) are well represented in the collections and could be productively researched by scholars. Of course Scotland, like every other country, had been ravaged by pestilence through the centuries where Edinburgh’s Old Town, with its famous closes and tenements, was a fertile environment for the spread of disease. Our special collections contain a number of fascinating accounts and sources for contagions in Scotland. Gilbert Skene’s *Ane Breve Description of the Pest* (Edinburgh, 1568)^1^ is one of the rarest items in our collection. One of the nation’s greatest manuscript survivals is that produced by George Bannatyne (1545–1608) during an outbreak of plague in 1568, into which he copied many vernacular poems, which would otherwise have been lost.^2^ He finished his manuscript with a personal poem, ‘Heir endis this buik writtin in tyme of pest / Quhen we fra labor was compeld to rest’. More recently, one of the most successful exhibitions mounted by the Library ran over 2014–2015 and was called ‘Plague! A cultural history of contagious diseases in Scotland’.^3^

Little did we know, that a mere 5 years after the exhibition, the Library would be closed until further notice, as the government exercised sweeping emergency powers that compelled us to stay at home. The volte-face in the United Kingdom took most of us by surprise. One moment we were musing on the effectiveness of ‘herd immunity’ and wondering why the New York Public Library and the Bibliothèque Nationale had closed the week before and the next moment we rapidly executed a plan and closed the Library on Friday, 20 March.

## Lockdown – Thinking on your feet

Quite how we managed to close the Library and move all of the staff off-site at such short notice is down to team working, leadership and everyone playing their part. An emergency can often bring the best out in people. It certainly focused the minds of everyone in the Library. A Business Continuity Team had been established early in March to monitor the unfolding situation and identify actions necessary to put enhanced safety measures and cleaning regimes in place.^4^ This focused on signage, planning revised layouts of the reading rooms, advising staff on the risks and mitigations and restricting footfall by cancelling events. The prospect of a total lockdown, closure of the Library and the government putting the economy into a coma was not seriously considered in those first weeks.

The first important date for the Library was the UK Prime Minister’s televised address on Monday, 16 March, in which he instructed ‘everyone’ to cease unnecessary contact with others and to stop all unnecessary travel. The public was to avoid ‘pubs, clubs, theatres and other such social venues’.^5^ It was also indicated that further advice would be issued in a few days directing that those with the ‘most serious health conditions’ were to shield for 12 weeks. For the Library’s plans, this was a defining moment as it gave us the main elements of how we would respond during that momentous week. The keywords and phrases were ‘unnecessary contact’, ‘unnecessary travel’, ‘avoid’ social venues and the heightened danger to those with underlying health conditions. All of these mapped over to the public who used our services and to the staff who provided them.^6^

The Library Lockdown Emergency plan set the framework for our response and, among other elements, made it clear how decision-making would flow.^7^ This was executed in conjunction with existing Business Continuity Plans with a tight relationship between the Business Continuity Team and the Library Leadership Team who met one after the other every day prior to the closure of the buildings.^8^ Communication was the essential underpinning to all of the planning with a daily ‘Librarian’s Update’ going out to all staff and a revised version going to the Library’s Board members at the end of each day. Communication was guided by the Librarian and Associate Director for External Relations, with support from the Head of Media.

Two crucial decisions were taken on Tuesday, 17 March. The first was to get off-site those staff with underlying health conditions and those with caring responsibilities. Staff in the publicised ‘at-risk’ groups were to discuss arrangements with their line managers including working from home, where possible. On Wednesday, 18 March, those instructed to make preparations to leave the Library included staff with school-age children (in anticipation of the closure of schools at the end of the week). These discussions had to be concluded at the latest by Friday, 20 March. At the time, we did not know exactly how many would leave each day, but we found enough capacity to manage down the Library through that week. Nevertheless, areas such as security and cleaning were substantially reduced within 24 h as relevant staff isolated for 12 weeks. None of our previous planning or data were able to predict this situation or the numbers of staff involved. The second crucial decision was to close the Library to the public the next day, at 5 p.m. Wednesday, 18 March.

Through that week and until the closure of the buildings, there was heightened guidance on avoiding face-to-face meetings, enhanced hygiene measures and maintaining social distance. At the same time, the plans to identify and prepare for a large-scale working from home programme were accelerated. The Digital teams executed a plan to rapidly move to an online only library and shifted the majority of the workforce to MS Teams and distributed about 50 extra laptops. In the second half of the week, attention focused increasingly on staff well-being and emphasising the need to stay in touch and support each other. A crisis response site on MS SharePoint was also created which held communications, well-being help, working from home guidance, training opportunities and projects to work on from home.

## The distributed library, or working from home

One of the truly positive outcomes from the pandemic is how it jolted us all into working from home and being effective away from the office. The crisis gave us no other option, so we had to make it work. The vast majority of staff took to it with typical verve and imagination. The instruction from the Librarian was to employ ‘best endeavours’, meaning work as well and productively as you can in the circumstances. This tended to ease concerns for those only able to do a limited amount of work.

Within a few weeks, the distributed library staff were holding hundreds of meetings each week on Microsoft Teams and were experimenting with Zoom, chat apps and Google resources. More immediately, the depletion of our staff through isolation reduced our on-site security detail which was boosted after a call for volunteers, while our Disaster Response and Recovery Team benefited from further volunteers coming forward.

Library staff were offering a remote enquiry and chat service to the public on Monday, 23 March, and this continues with further refinements and improvements such as additional operating hours. Monthly online workshops on Maps, family research and subscription resources were run by Readers’ Services staff while Curator-led talks at 5 p.m. on aspects of the collections regularly ‘sold out’. All in all, events moved online very quickly and proved popular on Zoom (the medium of choice) with larger and more geographically diverse audiences than were achieved in the Library.^9^ Promotion of resources targeting particular groups, such as children and families, was diligently pursued. Pleasingly, backlogs of digitised content waiting to be published were done from home. A highly popular example was the Moving Image Archive team’s efforts in Glasgow who loaded dozens of films online during lockdown, including a personal favourite about tattie howking in Ayrshire.^10^

An impressive range of initiatives were launched by staff which called for assistance across the Library. Programmes such as ‘Metadata From Home’ saw the transcription of a manual bookplate index and the updating of pre-1701 Scottish book records which were added to our National Bibliography of Scotland. The Library’s Mass Digitisation Team launched an OCR correction campaign using the Wikisource platform and were joined by over 60 staff members.^11^ This work enhanced the usability of our online Scottish collections.^12^ The general trend for usage of online services during lockdown was upward. Some spectacular increases included digital mapping services achieving 10,000–12,000 sessions per day and Moving Image Collections seeing hundreds of viewings of films each week. The Library’s social media presence had been growing over the last 6 years and this proved during lockdown to be our most effective medium for having conversations with the public and sharing links to resources.

Mood and morale, time management and focus all became different in lockdown. Feedback from staff and the results of a staff lockdown survey suggested that different teams would be up and down together. The daily updates from governments about infection rates, deaths and the dangers of leaving home must surely have contributed to the swings in morale from one week to another.

## Resumption planning

There are many contrasts between planning for resumption and planning for lockdown. At the time, going into lockdown seemed devilishly complicated and required a lot of fast decisions with minimal guidance from the Scottish government. Professional library discussion groups contained occasional helpful insights that were often obscured by a snowstorm of seemingly trivial comments and ‘quick surveys’. A lot of people were looking for reassurance that they were making the right decision which is, to a degree, understandable. Therefore, the Library closed to the public and vacated all of its buildings largely by reacting to the guidance available from government, adapting our business continuity plans and applying a large amount of common sense.

By contrast, resumption planning presents a range of problems across just about every aspect of the Library’s services. Guidance from government is provided on a regular basis, sectoral guidance is also supplied by government and Library agencies contribute information, surveys, toolkits, checklists and case studies. Going out there was too little guidance, going back in there is probably a little too much.^13^

Be that as it may, the Library’s approach is to use standard procedures for reoccupying buildings and gradually standing up services. Dates have been set for the resumption of public services in Edinburgh (11 August) with our Glasgow facility following closely behind. The initial offer will be limited and general occupation in reading rooms will be 25% of normal levels, and this will be the same for staff occupancy of buildings. Much like every other institution, risk assessments, health and safety issues, liaison with local trade union representatives and communication with the staff and public are all at the centre of planning and thinking.

## The new normal?

It is too early to say how much the experience of the coronavirus pandemic will change how the National Library of Scotland will function and serve the public. Making predictions when faced with dozens of decisions about resumption and budget planning perhaps results in poor predictions.

The human need to get back to normal and interact again with fellow citizens and colleagues, to come to a library as a place of learning and research (a safe space, in fact), will prevail. Many of us crave the smell of special collections in a reading room with spectacular views over Edinburgh, the sight of a well-curated exhibition and the delight of a cappuccino in the library cafe. Yet what may well change, or at least accelerate, is the use of digital resources, mixed office and homeworking and, of course, online meetings with long electronic agendas. The National Library of Scotland was already moving down that road at a growing pace and while our new strategy, ‘Reaching People 2020–2025’, is due to be published later in 2020, it was substantially written before Covid-19 silently upended our lives.

## Notes

1 Skene G (1568) *Ane Breve Description of the Pest*. Edinburgh. NLS Shelfmark: H.33.d.41.

2 National Library of Scotland (NLS), *Bannatyne Manuscript*, Adv.MS.1.1.6. Digital copy: https://digital.nls.uk/early-scottish-manuscripts/archive/74466548 (accessed 1 July 2020).

3 National Library of Scotland, ‘Plague! A cultural history of contagious diseases in Scotland’, exhibition, 2014–2015: https://www.nls.uk/exhibitions/plague (accessed 25 June 2020).

4 Before that our Human Resources colleagues were sending out email updates on Covid-19 to staff from 6 February 2020.

5 UK Government, Johnson B ‘Speech. PM statement on coronavirus: 16 March 2020’, https://www.gov.uk/government/speeches/pm-statement-on-coronavirus-16-march-2020 (accessed 25 June 2020).

6 It was most unfortunate that a very few managers decided to act immediately and either sent their staff home or left on Monday and did not return. The vast majority of staff, however, waited on guidance from the Librarian and the Leadership Team and followed an orderly sequence through the week.

7 This followed standard protocols and terminology, such as clearly laying out the governance flow – Strategic (Gold): Library Board, Librarian/CEO, Library Leadership team; Tactical (Silver): Business Continuity Team, Emergency Response Team – Resumption Plan, Salvage Plan, Response Plan; Operational (Bronze): Estates, Security, Cleaning, Health and Safety, Collections Support Services (CSS).

8 The Business Continuity Team (BCT) was led by the Operational Controller (Head of Estates). At various times it was unofficially called different things (e.g. Pandemic Response Team; Library Emergency Response Team) but the function remained essentially the same. The Library Leadership Team contained the Librarian, Director of Business Support, five Associate Directors covering major areas of collections and services and the Head of Human Resources.

9 National Library of Scotland, Library Player, https://www.nls.uk/player (accessed 25 June 2020)

10 National Library of Scotland, ‘Ayrshire Early Potatoes’ (1948), https://movingimage-onsite.nls.uk/film/2744 (accessed 25 June 2020).

11 Wikipedia, https://en.wikisource.org/wiki/Wikisource:WikiProject_NLS (accessed 1 July 2020).

12 See, for example, Scottish Chapbooks, https://digital.nls.uk/chapbooks-printed-in-scotland/archive/104184103, and then to our Data Foundry, https://data.nls.uk/data/digitised-collections/chapbooks-printed-in-scotland/ (accessed 25 June 2020).

13 This is an observation rather than a criticism: guidance, case studies and checklists are widespread and helpful, Scottish government, https://www.gov.scot/coronavirus-covid-19/; IFLA, https://www.ifla.org/covid-19-and-libraries; Research Libraries UK (RLUK), https://www.rluk.ac.uk/report-the-initial-responses-of-rluk-member-libraries-to-the-covid-19-crisis/ (accessed 1 July 2020).

